# Identifying ‘corridors of HIV transmission’ in a severely affected rural South African population: a case for a shift toward targeted prevention strategies

**DOI:** 10.1093/ije/dyx257

**Published:** 2017-12-28

**Authors:** Frank Tanser, Till Bärnighausen, Adrian Dobra, Benn Sartorius

**Affiliations:** 1 Africa Health Research Institute, Durban, South Africa; 2 School of Nursing and Public Health, University of KwaZulu-Natal, South Africa; 3 Centre for the AIDS Programme of Research in South Africa (CAPRISA), University of KwaZulu-Natal, Durban, South Africa; 4 Institute of Epidemiology and Health Care, University College London, London, UK; 5 Department of Global Health and Population, Harvard T.H. Chan School of Public Health, Boston, MA, USA; 6 Institute for Public Health, University of Heidelberg, Heidelberg, Germany; 7 Department of Statistics, Department of Biobehavioral Nursing and Health Informatics, Center for Statistics and the Social Sciences and Center for Studies in Demography and Ecology, University of Washington, Seattle, WA, USA

**Keywords:** HIV incidence, disease clustering, small-area variation, geographical information systems

## Abstract

**Background:**

In the context of a severe generalized African HIV epidemic, the value of geographically targeted prevention interventions has only recently been given serious consideration. However, to date no study has performed a population-based analysis of the micro-geographical clustering of HIV incident infections, limiting the evidential support for such a strategy.

**Methods:**

We followed 17 984 HIV-uninfected individuals aged 15–54 in a population-based cohort in rural KwaZulu-Natal, South Africa, and observed individual HIV sero-conversions between 2004 and 2014. We geo-located all individuals to an exact homestead of residence (accuracy <2 m). We then employed a two-dimensional Gaussian kernel of radius 3 km to produce robust estimates of HIV incidence which vary across continuous geographical space. We also applied Tango's flexibly shaped spatial scan statistic to identify irregularly shaped clusters of high HIV incidence.

**Results:**

Between 2004 and 2014, we observed a total of 2 311 HIV sero-conversions over 70 534 person-years of observation, at an overall incidence of 3.3 [95% confidence interval (CI), 3.1-3.4] per 100 person-years. Three large irregularly-shaped clusters of new HIV infections (relative risk = 1.6, 1.7 and 2.3) were identified in two adjacent peri-urban communities near the National Road (*P* = 0.001, 0.015) as well as in a rural node bordering a recent coal mine development (*P* = 0.020), respectively. Together the clusters had a significantly higher age-sex standardized incidence of 5.1 (95% CI, 4.7-5.6) per 100 person-years compared with a standardized incidence of 3.0 per 100 person-years (95% CI, 2.9-3.2) in the remainder of the study area. Though these clusters comprise just 6.8% of the study area, they account for one out of every four sero-conversions observed over the study period.

**Conclusions:**

Our study has revealed clear ‘corridors of transmission’ in this typical rural, hyper-endemic population. Even in a severely affected rural African population, an approach that seeks to provide preventive interventions to the most vulnerable geographies could be more effective and cost-effective in reducing the overall rate of new HIV infections. There is an urgent need to develop and test such interventions as part of an overall combination prevention approach.

## Introduction

Despite impressive life expectancy gains due to antiretroviral treatment,^1^^,^^2^ the rate of new HIV infections remains high in many communities in sub-Saharan Africa.^3^^,^^4^ Consequently, innovative approaches are needed for prevention strategies that make better use of the limited resources available. Implementing effective HIV prevention programmes requires knowledge of the geographical distribution of HIV incidence and risk factors associated with acquisition of infection.^5^ However, the value of geographically targeted prevention interventions has only recently been given serious consideration in the context of a severe generalized African epidemic. In this regard, international agencies such as Joint United Nations Programme on HIV/AIDS (UNAIDS)^6^ and the Global Fund^7^ have recently recommended that countries move to adopt a geographical prioritization approach to optimize the provision of HIV prevention and treatment services. A particularly prominent adopter of this approach is the United States President's Emergency Plan for AIDS Relief (PEPFAR) which seeks to use a ‘data-driven approach that strategically targets geographic areas and populations where we can achieve the most impact’.^8^ This approach allocates resources on the basis of a geographical prioritization of districts, focusing on those geographical regions and localities where most transmission is occurring. The strategy recognizes that targeting the populations implicated in these sub-epidemics is vital to achieving large reductions in population-level incidence.

This recent shift in thinking has been driven by high levels of HIV prevalence in some communities,^9–11^ detection of localized spatial clustering of prevalent cases and HIV-related deaths,^12–15^ variation in HIV prevalence at a district and clinic level^16^^,^^17^ and geographical variation in HIV incidence among participating women in clinical trials.^18^ These results have challenged the previous paradigm of a ubiquitous ‘generalized’ epidemic in many hyper-endemic contexts. Furthermore, mathematical models^19^ and phylogenetic research^20^^,^^21^ have suggested that there may be real prevention gains to be made by harnessing geographical differences in HIV incidence for epidemic control, by intervening aggressively in the most vulnerable, high-risk populations. However, to date there remains limited population-based evidence from a hyper-endemic setting to demonstrate whether incident HIV infections do indeed cluster in space and therefore whether a geographically targeted strategy could potentially pay prevention dividends. Such evidence is difficult and expensive to obtain because of the large sample sizes and long follow-up times required to directly observe a sufficient number of HIV sero-conversions so as to be able to provide robust statistical inference when quantifying geographical variations in HIV incidence. If this type of population-based evidence were forthcoming, it could provide real impetus to more widely adopt a strategy that seeks to deploy specific interventions in high-risk geographical spaces as part of an overall combination prevention approach.

Given this background, we followed up nearly 18 000 HIV-uninfected individuals (observing individual HIV sero-conversions) over a decade, in a typical hyper-endemic, rural South African setting. We precisely geo-locate all participants to an exact homestead of residence and use advanced spatial analytical techniques to identify and characterize micro-geographies with excessive numbers of new HIV infections.

## Methods

### Setting

The study uses data from one of the most comprehensive demographic surveillance sites in Africa—the Africa Centre (now Africa Health Research Institute) Demographic Information System. ^22^ The site has collected sociodemographic information on a population of approximately 87 000 individuals within a circumscribed geographical area (438 km^2^ in area) in rural KwaZulu-Natal, South Africa, for over a decade. One of the notable strengths of the comprehensive demographic platform is its longitudinal integrity and ability to record exact periods of time spent living at multiple locations (including outside the study area) by each individual under surveillance.^22^ Nested within the demographic information system are the population-based HIV surveillance and sexual behaviour surveys which take place annually. Between 2004 and 2006, all women aged 15–49 years and men aged 15–54 years resident in the surveillance area were eligible for HIV testing. However, starting in 2007, eligibility was extended to cover all resident individuals’ ≥ 15 years of age. The longitudinal dynamics of participation in the HIV survey is described in detail elsewhere.^23^ Overall, 29% of the adult population aged15 49 are infected with HIV.^24^ The rate of new HIV infections is high at around 2.7 new infections per 100 person-years in the entire adult population (≥ 15 years of age).^4^

Ethics approval for all surveillance data collection activities was obtained from the Nelson Mandela Medical School Research Ethics Committee, University of KwaZulu Natal, Durban.

### Statistical analysis

Individuals (aged 15–54) were included in the analysis if they tested negative for HIV upon entry into the surveillance cohort and consented to test at least once thereafter (*n* = 17 984); 80% of participants in the cohort agreed to an HIV test at their first test offer, and 62% observed to be HIV-negative at one point in time were tested on at least one subsequent occasion. Participants seldom test every year, and the median interval of time between last HIV-negative and first HIV-positive test is 2.18 years. The date of HIV seroconversion was assumed to occur according to a uniform random distribution between the date of the last negative and first positive HIV test. This avoided the biases introduced by assuming that the date of HIV sero-conversion occurred at the mid-point of these two dates.^25^ To investigate the age-sex trends in incidence, we computed HIV incidence [95% confidence interval (CI)] by 5-year age-band. Crude incidence rates per 100 person-years were age-sex standardized against the age and sex structure of the entire cohort for the full period. We also computed incidence by 1-year age band (stratified by gender) and fitted a log-normal function for each group using a maximum likelihood approach.

### Spatial analyses

We geo-located all participants to their respective homesteads of residence which have been comprehensively mapped to an accuracy of < 2 m.^14^ The population is characterized by a high level of mobility,^26^ and the demographic information system was set up so as to be able to precisely measure the amount of time each individual was resident at any given location within the study area.^22^ To ensure unbiased geographical estimates of incidence, it is important that we adequately account for the mobility in the study population and do not impose an overly simplistic static residency assumption on the data. To accomplish this, we included in the incidence denominator only person-days of exposure that accrue to participants while resident in the study area (i.e. person-days spent outside the study area were not included in the incidence rate calculations). Similarly, if the imputed sero-conversion date coincided with a period of time during which the participant lived outside the study area, then the incident event was not included in the numerator. In cases where a participant was resident at multiple homesteads during the period of observation, the exact number of days spent at the location of each homestead was used in the geographical analyses. Incident events were attributed to the location of the homestead at which the participant was resident at the imputed sero-conversion date. This approach ensured that the numerator and denominator in the incidence calculation were not systematically biased in either direction, without the need to super-impose an arbitrary residency criterion or assume that individuals were resident at a single location between survey rounds. We then used two different spatial analytical techniques to measure the spatial distribution of HIV incidence across the study area; these are described in detail below.

First, we used a Gaussian kernel (radius = 3 km) methodology to produce robust estimates of HIV incidence that vary across continuous geographical space.^14^^,^^27^ The size of the kernel was determined on the basis of previous work in this population, which measured the spatial dependence in ward-level HIV prevalence.^14^ The smaller the radius used in the kernel, the greater the range in estimates obtained and the greater the sensitivity to local variation. The use of a larger kernel will result in smoothing towards the mean, and important variation in HIV incidence may be lost. The Gaussian kernel does not impose any static geographical boundaries on the data, but uses the precise location of each individual to derive the resulting community-level estimates. A median of 463 [interquartile range (IQR): 211-1033] person-years of observation was evaluated in the virtual community surrounding each participant’s homestead in the kernel-based approach. The methodology produces spatially continuous HIV incidence estimates that are sensitive to local variations while at the same time being robust to the effects of random noise.

From a resource allocation perspective, it is important to quantify not only the risk of new HIV infection over person-time but also the density of new HIV infections per unit area. In our previous work, we used the Gaussian kernel approach to estimate the density of existing HIV infections per km^2^.^14^ In a similar fashion, to derive the density of HIV incident events, we multiplied the HIV incidence map by the geographical distribution of all HIV-negative residents in 2014 (adjusted for non-consent) to derive the total number of HIV sero-conversions per km^2^ per year. The latter surface was derived from the 2014 population-based HIV sero-survey of 9508 individuals living in the study area. We applied an edge correction factor within 3 km of the boundary of the study area, to accurately quantify the total number of HIV sero-conversions per km^2^ across the whole study area in a given year.

Second, for the first time to our knowledge, we applied Tango's flexibly shaped spatial scan statistic^28^ (implemented in FleXScan software^29^) to identify micro-geographical clustering of new HIV infections. Spatial scan statistics are designed to detect a local excess of events and to test whether such identified excess could reasonably have occurred by chance.^30^ A window is imposed on an area of interest by the statistic, and the centre of the windows moves across the study region while also varying in size (diameter). The spatial scan statistic calculates the likelihood of observing the number of events inside and outside each window. The window with maximum likelihood is defined as the most likely cluster, i.e. least likely to have occurred by chance. Most disease or health outcome events are not likely to conform to a predefined geometrical shape (circle or ellipse) as imposed by other commonly used spatial scan statistics, such as the Kulldorff statistic.^31^ The Tango flexible spatial scan statistic instead allows an irregularly shaped scanning window to be imposed on each location by iteratively adding connected (or adjacent) locations. Therefore, this advanced analytical technique can detect irregularly (arbitrarily) shaped spatial clusters by iteratively (via Monte Carlo replication) combining adjacent locations, and has higher power when the true cluster is non-circular compared with a regularly shaped spatial scan statistic.^28^^,^^32^

We assumed a Poisson model for these analyses, i.e. number of incident events (numerator) scaled by person-years (PY) as the denominator. The Poisson model calculates an expected number of incident events based on the overall incidence rate, and applies this to the observed denominator (PY) within a given scan window (‘cluster’) to calculate the expected number of events. The ratio of the observed vs expected yields the relative risk (RR) for a given cluster. A *P*-value for the observed cluster (difference) is also calculated, based on the null distribution of likelihood ratio test statistic with a large number of Monte Carlo replications of the data set generated under the null hypothesis.^28^ We classified any cluster with a *P*-value of < 0.05 as being statistically significant.

More specifically, consider the scenario where the study area is divided into m nodes (∼800 aggregated homesteads as specified above). The number of cases in a given node *i* can be represented by the random variable *N_i_* with observed value of incident HIV cases *n_i_*, where *i = 1*, …, *m.* Under the null hypothesis (H_0_) of no clustering, the *N_i_* are assumed to be independent Poisson variables whereby:

$$H_{0}:\;E(N_{i})=\xi_{i}\;,\;\;N_{i}∼\;\text{Pois}(\xi_{i}),\;i=1,\;\ldots ,\;m$$

We employed the likelihood ratio (LLR) with restriction statistic by Tango (2005)^28^ as implemented in FleXScan, with a default restriction (‘alpha’) parameter setting of 0.2. This approach avoids detecting meaninglessly large clusters (little practical/policy relevance in a relative small geographical area) and also improves calculation time with a large number of locations, as found in our data. Furthermore, the pre-specified maximum length of cluster (or K parameter) for the flexible spatial scan statistic has to be set at a realistic upper bound to avoid computationally infeasible scans. The current practical upper bound as suggested by Tango is around K = 30, which we employed in our analyses.^28^ Tango has suggested that the execution time of the current algorithm will take more than a week if K > 30 for the number of regions m ∼200–300. However, in our data we had 8590 unique locations before aggregation. With a value of K = 30 it would be statistically impossible to identify significant and meaningful clusters, as the scan statistic would only search through a maximum of 30 adjacent nodes (homesteads), and the number of incident HIV events in such a number of homesteads would be largely 0 across the site. Hence our choice to aggregate the homesteads to a regular grid of *n* = 760, for the FleXScan analysis to be able to identify meaningful clusters with significance without substantial loss of fine geographical precision/resolution. 

### Cluster characterization

We employed mixed effects linear and logistic regression models to compare selected characteristics between clusters and non-clusters. The regression models include fixed effects for cluster membership and normally distributed random intercepts that capture the correlation between the samples associated with the same individual. Testing whether there is a relationship between a selected characteristic and the clustering of locations is done by testing the null hypothesis, that the fixed effects for cluster membership are zero, against the alternative hypothesis, that at least one of the fixed effects is different from zero (see Supplementary materials, available as Supplementary data at *IJE* online).

We examined the relationship between incidence rates and the clustering of locations by fitting separate survival models for men and women. These are Cox proportional hazards models for time to HIV seroconversion, which have cluster membership indicator variables as time-dependent covariates. Assessing whether the clustering of locations is associated with the hazard of HIV acquisition is performed by testing the null hypothesis that the slope coefficients associated with cluster membership are zero. A complete description of the statistical models employed is provided in the Supplementary materials, available at *IJE* online. 

## Results

**Figure 1:**
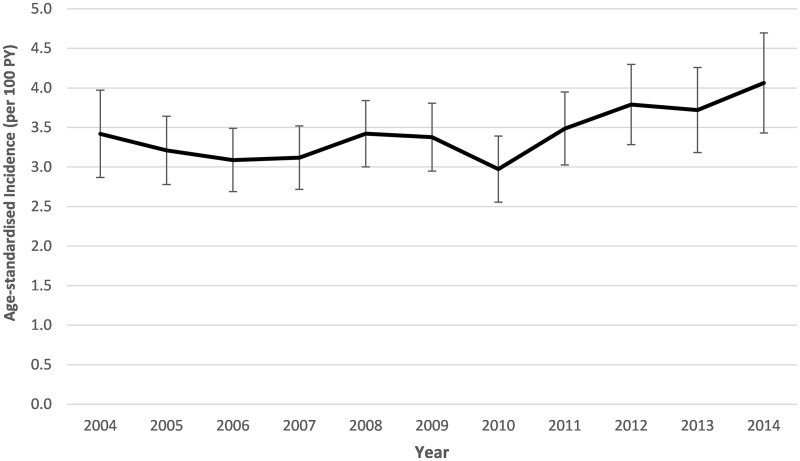
Age-sex standardized HIV incidence by year for individuals aged 15–54 [70 534 person-years (PY), 2311 sero-conversions at crude incidence of 3.3 per 100 PY].

**Figure 2:**
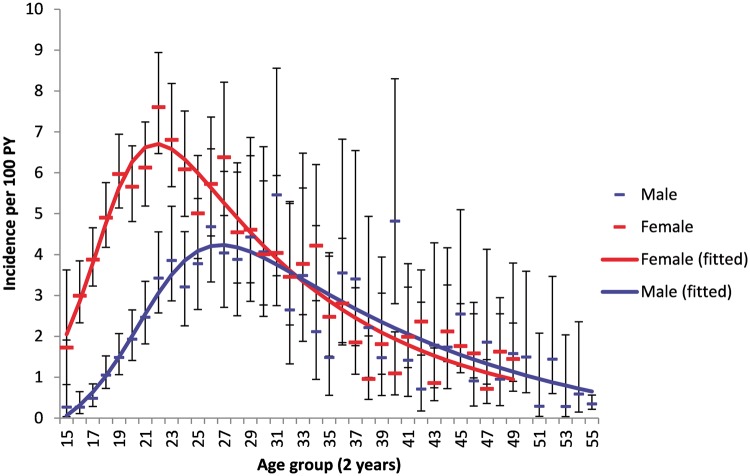
Female and male age variations in HIV incidence (95% CI) for entire sample of repeat-testers aged 15–54. Superimposed on the graphs are log-normal functions (obtained by maximum likelihood) fitted to 1-year incidence estimates.

Between 2004 and 2014, we observed a total of 2311 HIV sero-conversions during 70 534 person-years of observation among the cohort, at a crude HIV incidence rate of 3.3 (95% CI, 3.1–3.4) per 100 person-years. The overall HIV incidence rate amongst the male population for the period was 2.0 per 100 person-years (95% CI, 1.8–2.2) compared with 4.1 per 100 person-years (95% CI, 3.9–4.3) among the female population. Overall incidence for the population remained relatively stable when assessed by year (Figure 1). Based on a log normal curve (Figure 2), incidence was highest in females 22 years of age at an incidence of 7.6 cases per 100 person-years (95% CI, 6.5–8.9) and peaked later in males at 27 years of age (4.0 incident cases per 100 person-years, 95% CI, 2.7–6.0). Incidence risk for females was much higher at earlier age but inverted at ∼32 years of age, when males’ risk was subsequently marginally higher. The striking sex differences in HIV incidence are a result of a combination of increased biological susceptibility to infection,^33^ as well as the unequal cultural, social and economic status of (particularly young) women in society.^34^^,^^35^

**Figure 3:**
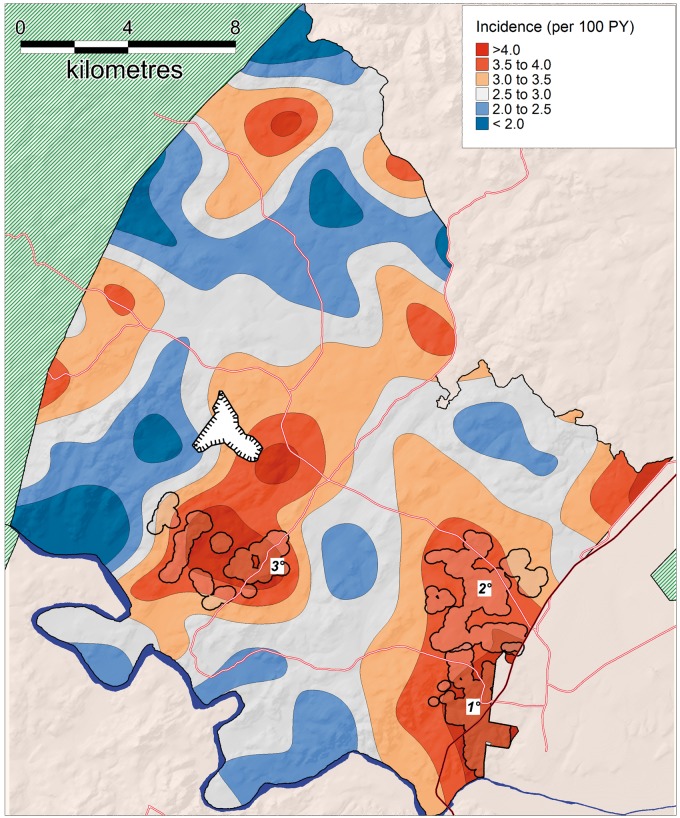
Geographical variations in population-level HIV incidence (ages 15–54) as measured by a standard Gaussian kernel (3.0 km radius). Superimposed on the map are the high-risk clusters identified by the Tango's flexibly shaped spatial scan statistic: Cluster 1 [322 sero-conversions, 6233 person-years of observation (PYO), RR = 1.59, *P* = 0.001, area = 8.3 km^2^]; Cluster 2 (185 sero-conversions, 3630 PYO, RR = 1.57, *P* = 0.015, area = 12.7 km^2^); and Cluster 3 (64 sero-conversions, 891 PYO, RR = 2.27, *P* = 0.020, area = 9.4 km^2^). Phase 1 of a recent opencast coal mining development is shown immediately north of cluster 3.

**Figure 4:**
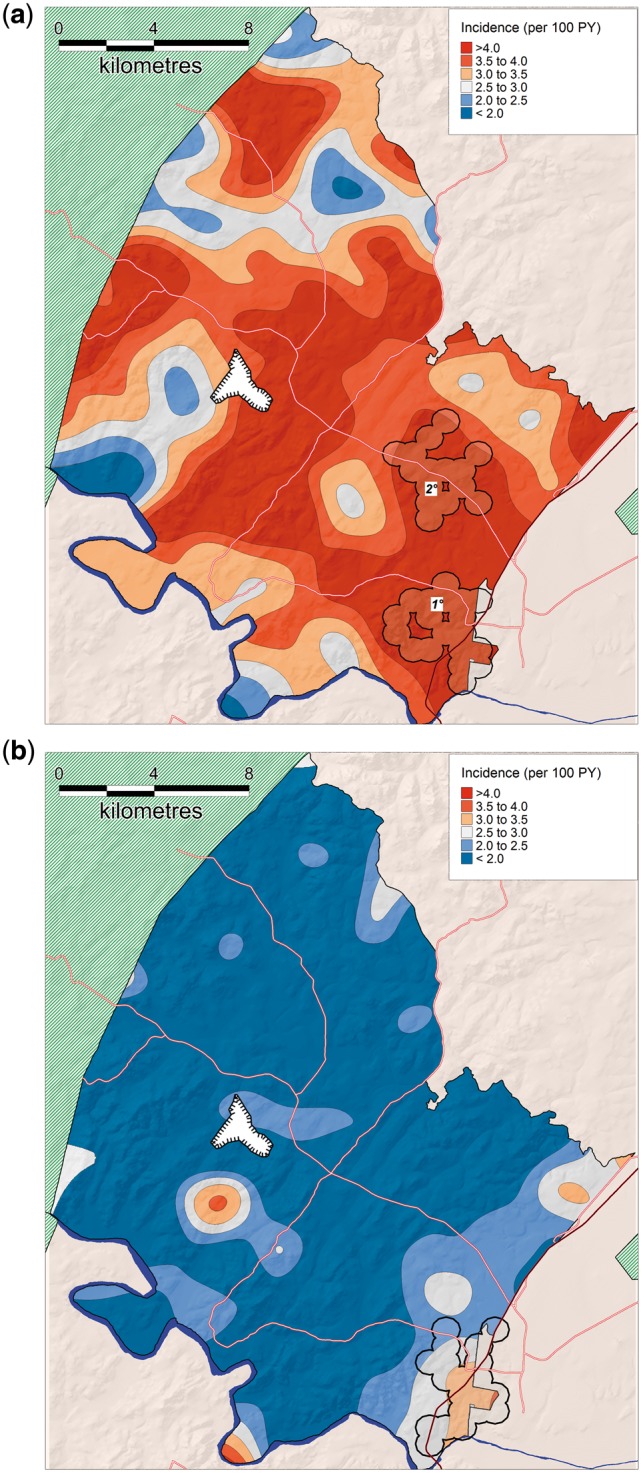
Geographical variations in population-level HIV incidence (ages 15–54) in females (a) and males (b), as measured by a standard Gaussian kernel (3.0 km radius). Superimposed on the map are the high-risk clusters identified by the Tango's flexibly shaped spatial scan statistic: (a) Cluster 1 = 296 sero-conversions, RR = 1.52, *P* = 0.002; Cluster 2 = 91 sero-conversions, RR = 1.57, *P* = 0.081; and (b) Cluster = 131 sero-conversions, RR = 1.92, *P* = 0.001.

Our analyses revealed considerable spatial variation in HIV incidence (Figure 3). Overall, the kernel-based spatial analytical approach showed that HIV incidence varied between 0.86% and 5.21% per year in the unique virtual community evaluated around each participant’s homestead (a > 6-fold variation). Three clear high-risk spatial clusters (RR = 1.6, 1.7 and 2.3, respectively) were identified using the flexibly shaped spatial scan statistic (Figure 3). Together the clusters had a significantly higher standardized incidence of 5.1 (95% CI, 4.7–5.6) per 100 person-years compared with a standardized incidence of 3.0 per 100 person-years (95% CI, 2.9–3.2) in the remainder of the study area. Clusters 1 and 2 (4.9 and 4.1 km in extent, respectively) were in peri-urban communities in the south-east and eastern portion of the study site, both in close proximity to a National Road (*P* = 0.001, 0.015, respectively). The third cluster (4.7 km in extent) was situated more internally to the south central of the study area, near a recent coal mine development which first became operational in 2007 *P* = 0.020). The clusters contained 322, 185 and 64 incident cases, respectively (25% of all incident cases observed over the duration of the study). The clusters had standardized incidence rates of 5.3 (95% CI, 4.7–5.8), 4.6 (95% CI, 3.9–5.3) and 6.7 (95% CI, 4.9–8.4) per 100 person-years, respectively, for the period 2004–14. Whilst the HIV incidence in clusters 1 and 2 (peri-urban communities) remained relatively constant over time, cluster 3 (located near the coal mine) showed a striking increase in standardized HIV incidence (per 100 PY) towards the end of the study period, from 6.1 (2004–08) to 5.6 (2009–12) to 16.2 between 2013 and 2014 (time period 3 vs time period 1 hazard ratio = 3.34, *P* = 0.001). Analysis of spatial clustering of HIV incidence stratified by gender suggested a similar high-risk cluster respectively for both females and males in the the same location as the primary cluster identified as part of the combined sex analysis, bordering the national highway (Figure 4a, b). Similarly, there was some evidence of a secondary female cluster (P=0.086) in a similar location to the secondary cluster identified as part of the combined sex analysis.

Overall, the most prominent differences in the cluster vs non-cluster communities (Figure 3) were the population growth rate between 2004 and 2014 (+6.1% vs -10.9%), migration intensity (160 vs 148 migration events per 100 person-years) and HIV prevalence (38.9% vs 27.5%) (Table 1). The cluster communities also had higher numbers of reported lifetime partners, lower reported number of years of education and lower reported mean age at first sex (females). There was a lower number of births per 1000 females of child-bearing age, and contraceptive use was marginally higher in the cluster communities. Overall, the ratio of female to male incidence was 14% lower (i.e. male incidence was disproportionally higher) in the cluster communities (compared with non-clusters) but this difference was not significant (*P* = 0.185).

**Table 1:** Comparison of key characteristics of three high-incidence clusters identified by the Tango flexible spatial scan-statistic (Figure 3) vs non-clusters

Characteristic	Non-cluster (*n* = 15254)	Cluster (*n* = 2730)	*P*-value^a^
% Female	58.9	58.5	0.122
Ratio of female to male incidence^b^	2.2	1.9	0.185
Mean (SD) age of adults (15–54 years)	28.0 (11.3)	28.3 (10.8)	**< 0.001**
Median (IQR) number of lifetime partners (females)	1 (1-2)	2 (1-2)	**< 0.001**
Mean (SD) years of education of adults (15–54 years)^b^	9.2 (3.6)	8.8 (3.5)	**0.007**
Household assets, mean (SD) quintile score	3.0 (1.4)	2.9 (1.4)	**< 0.001**
Fertility rate (births per 1000 females, 15–49 years, PY)^b^	62.5	62.2	**0.014**
Migration events among ages 15–54 years (per 1000 PY)^b^	147.8	160.6	**< 0.001**
Mean (SD) age first married (females)	26.1 (7.0)	27.4 (7.3)	**0.015**
Mean (SD) age at first sex (female)	18.0 (3.0)	17.7 (3.1)	**< 0.001**
Current contraception use (females 15–49 years) (%)^b^	44.0	46.9	**0.030**
Growth over study period (%)^c^	−10.9	+6.1%	**< 0.001**
HIV prevalence^d^ among adults (15–54 years) (%)	27.5	38.9	**< 0.001**

SD, standard deviation; IQR, interquartile range. Bold indicates a P-value of <0.05.

a Adjusted for within-subject correlation (multiple or repeated measurements). See supplementary materials for more details of the modelling approaches used, available at *IJE* online.

b Age-standardized.

c Based on growth of entire population under surveillance.

d Based on population-based HIV testing survey of 2014.

**Figure 5:**
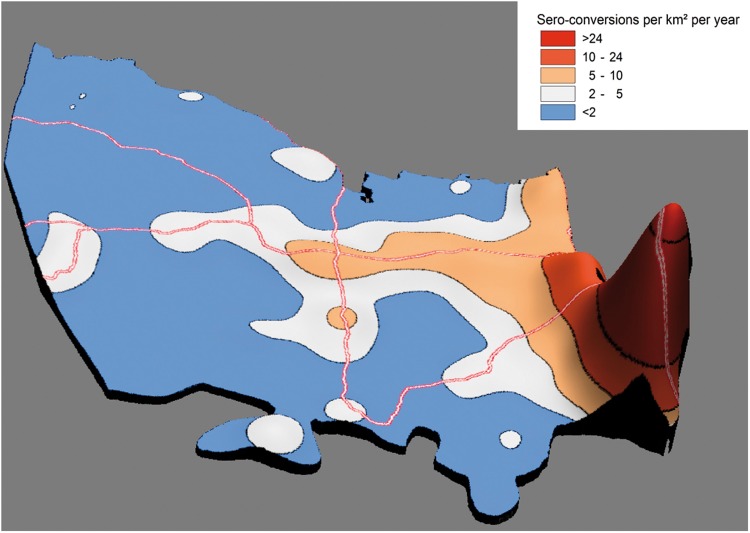
Estimated HIV sero-conversions per km^2^ per year for population aged 15–54, obtained using the Gaussian kernel (radius = 3.0 km). The Z axis is proportional to the total HIV sero-conversions per km^2^ per annum for any geographical location.

The map showing the estimated number of HIV sero-conversions per km^2^ per year is presented in Figure 5. Despite the clusters comprising just 6.8% of the study area, they account for one out of every four HIV sero-conversions observed over the study period. Of course, the ‘Achilles heel’ of such a geographically focused strategy is the detailed ‘granular’ data needed to be able to pin-point areas most in need of intervention. In most cases, such data are not available nor indeed feasible to collect. Given this reality, we quantified the implications of a more typical situation in which programme managers did not have knowledge of the exact location of the high-risk clusters, and were instead to target communities within 2 km of the National Road, which are characterized by both overall high incidence as well as a high population density (Table 2). Given this scenario, by focusing efforts on the 8% of the study area that lies within 2 km of the National Road, ∼37% of HIV sero-conversions observed could theoretically be targeted at an ‘effectiveness ratio’ (ratio of the proportion of sero-conversions targeted to proportion of the area covered) of 4.6.

**Table 2:** Hypothetical targeting strategies to evaluate the number of HIV sero-conversions relative to the size of a given geographical area

	Area km^2^ (% total)	Person-years (% total)	HIV sero-conversions (% total)	Effectiveness ratio^a^
High-risk clusters^b^	29.9 (6.8%)	10763 (15.2%)	550 (23.8%)	3.5
Communities within 2 km of National Road	35.8 (8.2%)	22641 (32.1%)	866 (37.5%)	4.6
Study area	438.1 (100%)	70534 (100%)	2311 (100%)	1.0

a The ratio of % sero-conversions targeted to % area covered

b Only high-risk clusters identified by the Tango flexible scan-statistic (Figure 3) targeted.

## Discussion

We have used one of Africa's largest ongoing population-based cohorts to analyse the micro-geographical clustering of HIV incident infections in a hyper-endemic, rural sub-Saharan African context. We use the precise location of each of the nearly 18 000 HIV-uninfected individuals (and exact time spent at each location) followed prospectively over a decade to reveal >6-fold geographical variations in HIV incidence. In addition to the ‘expected’ high-risk clusters in high-density populations near the National Road, the most surprising aspect of the analysis has been the rapid emergence of a rural high-risk cluster with unusually high numbers of new HIV infections near a recent mining development. Overall, the results provide clear empirical evidence for localized ‘corridors of transmission’, and imply that targeting evidence-based interventions to the most vulnerable populations in areas of greatest HIV incidence could be powerful and cost-effective in this typical rural African population, as part of an overall combination prevention approach. Our results add to an increasing body of work from multiple sub-Saharan African environments that have challenged the previous paradigm of a ubiquitous generalized epidemic.^9–11^^,^^14^^,^^15^^,^^18^^,^^36^ Rather, the findings demonstrate the existence of multiple geographically-defined sub-epidemics which make up the composite epidemic in a given population. Taken together, this body of work suggests that there may be a remarkable opportunity to exploit these geographical heterogeneities for epidemic control by intervening in the most vulnerable populations.

Significant clustering of new HIV infections in particular communities can decrease the efficacy of existing population-based intervention measures, but also implies that targeted interventions could be highly effective.^19^^,^^37^ Identification of the localized clustering of HIV incident infections, or so-called ‘hot spots’, also has important local policy relevance, because it has immediate implications for where to focus new prevention programmes or to intensify existing programmes.^38^ A recent review of research conducted in the 10 highest HIV prevalence countries in the world, concluded that ‘recent empirical findings combined with evidence from phylogenetic studies and supported by mathematical models provide a rationale for testing the feasibility, acceptability, and effectiveness of targeted HIV prevention approaches in hyper-endemic populations to supplement measures aimed at the general population’.^39^ Although some critical questions remain around the reach and acceptability of such interventions, the results of the review suggested that maximum reductions could be achieved by employing an approach that specifically seeks to target the most vulnerable geographies, in addition to a broad set of interventions targeting the larger population.

Our results raise a fundamental and a related practical question: what is the underlying reason for the clustering of these new HIV infections? High levels of mobility, high rates of sexual partner turnover, younger age at first sex and a high population growth rate are some of the key factors that have been implicated in this research. In recent work, based on the underlying theory of social disequilibrium,^40^^,^^41^ we investigated the individual, social and community challenges to HIV acquisition risk in this study population.^42^ We found that particularly among men, the effect of migration intensity in predicting HIV acquisition risk was more pronounced and localized in the high-incidence communities near the National Road. This finding was over and above the risk conferred on the basis of individual mobility levels, highlighting the multiple impacts of mobility on HIV acquisition risk and also attesting to the importance of the social and geographical context in predicting risk of HIV acquisition. These risk factors are particularly pertinent in South Africa, given the labour migration history in the apartheid era as well as extremely high levels of socioeconomic inequality.^43^^,^^44^ In addition, high concentrations of sex workers around mines and other industrial developments have been documented,^45^^,^^46^ and this constitutes one possible contributory factor in explaining the high incidence in the high-risk cluster adjacent to the coal mine. Ultimately however, these clusters arise from a complex web of structural, biological and sociodemographic factors which act together synergistically at the level of the individual, the network and the community to produce incidence rates of this magnitude. These factors have been the subject of research in our team and many others over the past 20 years.^26^^,^^47–49^ In the long term, if we more fully understand the underlying causal processes, we will be better placed to design and implement robust interventions across different contexts. Nevertheless, whereas a better understanding this web of causality remains of fundamental importance, basic characterization of the communities in a way that will allow them to be readily identified in other settings is of immense immediate value.

Despite the accuracy and strengths of the micro-geographical approach used, our work has some limitations. The length of time between last HIV-negative and first HIV-positive test in this cohort (≈2 years), combined with high levels of mobility in this population,^26^ mean that any geographical clustering analysis would be biased towards the null hypothesis of spatial randomness. Although these effects cannot explain the false detection of a spatial cluster of new infections, it is nevertheless conceivable that we may have missed detection of some clusters as a result. In addition, the analytical approach used in comparing clusters with non-clusters, in terms of the sociodemographic and behavioural characteristics, is ecological in nature and as such cannot be used to establish causal relationships. Work is ongoing to establish what drives spatial differences in risk, using individual-level spatial models to provide a stronger basis for causal inference.

In this typical rural setting, nearly 40% of all HIV sero-conversions take place in communities occupying only 8% of the study area. The combination of highest individual-level risk of infection as well as high population densities in these communities give rise to this finding. Thus, there will likely be efficiency gains in the deployment of prevention interventions to such communities, driven by economies of scale. Such gains would be further amplified if individuals in these communities play a disproportionate role in re-seeding epidemics in other populations or act as short-term ‘nodes of attraction’ for high-risk, HIV-infected individuals from more rural communities.^15^^,^^16^ In this regard, our results suggest that high-density peri-urban communities with rapid population growth, located along National Roads, should be prioritized for intervention in this and other similar settings. Given the high levels of mobility in these populations,^26^ it is likely that the effect of such an intervention would extend well beyond the intervention community. Similarly, populations surrounding recent mining and other industrial developments should be given special prevention consideration over and above the clear need to focus prevention efforts on the employees of these developments themselves. This may be particularly pertinent given the scale of the mining industry in South Africa and Southern African in general.^50^

The cornerstone of any combination prevention approach must be treatment as prevention.^51^ One obvious component of the prevention strategy, would therefore be to rapidly increase antiretroviral therapy (ART) coverage in these high-risk communities, particularly in light of attaining the UNAIDS 90-90-90 treatment targets.^52^ Remarkably, in some of the high-incidence communities near the National Road, 65% of HIV-infected individuals had unsuppressed viral loads and > 20% of the entire adult population—i.e. irrespective of HIV status—were viraemic for HIV in 2011 (7 years after roll-out of ART).^53^ We have previously demonstrated, in this real-world setting, that individual HIV acquisition risk declines significantly with increasing ART coverage in the local community,^4^ the household^54^ and the sexual partnership;^55^ and in forthcoming work, we show a significant population-level decrease in HIV incidence in men (consistent with a higher uptake of ART in women,^53^ as well as increase in the prevalence of circumcision).^56^ However, uptake of ART is impeded by geographical and transport-related barriers which can also produce negative HIV treatment outcomes in those individuals who have been initiated on ART.^57^^,^^58^ In this vein, we have shown empirically that there is a steep and immediate fall-off in uptake of ART with increasing distance from a service delivery point.^59^ For example, holding other factors constant, at approximately 4.8 km from a clinic providing ART, the odds of an HIV-positive individual being on ART are half those of someone living immediately next to a clinic. It follows that by locating ART services in these populous areas of highest transmission intensity, the most vulnerable populations could be intensively targeted to achieve maximum reductions in HIV incidence. Other components of such a strategy could include intensive messaging campaigns,^60^ the use of micro-financial incentives to increase rates of HIV testing and linkage to care particularly among men,^61–63^ improved access to voluntary medical male circumcision^64^ and the use of pre-exposure prophylaxis^65^ among vulnerable groups such as young women and sex workers^66^ (who may be over-represented in communities characterized by intense transmission).

Implementing a geographically orientated intervention approach is not without its drawbacks and programmatic challenges even when the epidemiological rationale is clear.^67^ First, the data required to implement the strategy can be numerous and the burden on health care workers to collect this data could be substantial. However, as we outlined earlier in the paper, even targeting ‘obvious’ high-risk populations, such as peri-urban communities living within 2 km of major transport routes, could achieve substantial prevention dividends. Second, there could be a danger that populations outside the high-risk communities could become marginalized in terms of treatment and prevention efforts. After all, 75% of HIV sero-conversions in this population still occur outside the identified incidence clusters, and individuals in these other communities are also vulnerable to HIV infection. It is therefore essential that good services continue to be provided to these populations as well, if the UNAIDS 90-90-90 targets are to be reached and the tide of the epidemic is to be turned through a sustained combination prevention approach. Third, scaling up services in high-risk communities implies a level of budget flexibility that may not exist in some HIV hyper-endemic contexts. However, notwithstanding these difficulties, the potential rewards of such an approach may be substantial. For example, a recent modelling exercise undertaken in Kenya estimated that, with no additional cost, a geographically tailored approach could result in a 33% drop in the rate of new infections towards the end of a 15-year period.^19^ A subsequent study estimated the potential impact of a localized, integrated approach to HIV prevention funding that prioritized populations on the basis of both geographical and individual risk factors, across the continent of Africa. The results suggested that for a US$20 billion representative expenditure over a 15-year period, scale-up of prevention along present funding channels could avert 5.3 million new infections, relative to no scale-up.^68^

Our study has revealed remarkable geographical variation in HIV incidence in this hyper-endemic population, with the existence of clear ‘corridors of transmission’ where the rate of new HIV infections was 70% higher than in surrounding communities. Targeting efforts at settings where HIV transmission is most intense is crucial. A considerable body of evidence now supports the contention that even in a severely affected rural African setting, interventions that strategically target geographically defined high-risk communities, as part of a combination prevention approach, could be more effective in reducing the overall rate of new infections. Most recently, this type of geographical prioritization approach has been adopted by PEPFAR in order to maximize the impact of their investment.^8^ Despite programmatic and other challenges, our empirical results, combined with insights from recent mathematical modelling studies as well as observations from other generalized epidemic settings, strongly suggest that, given finite (and likely decreasing) resources, targeted HIV prevention strategies could be effective even in a population with very high overall HIV incidence. There is therefore an urgent need to develop and test such interventions as part of an overall combination prevention approach. 

## Supplementary Data

Supplementary data are available at *IJE* online.

## Funding

This work was supported by National Institute of Health (R01HD084233 and R01AI124389) and South African Medical Research Council Flagship (MRC-RFA-UFSP-01–2013/UKZN HIVEPI) grants as well as a UK Academy of Medical Sciences Newton Advanced Fellowship (NA150161). AD was supported in part by the National Science Foundation Grant DMS/MPS-1737746 to University of Washington. TB was supported by the Alexander von Humboldt Foundation through the Alexander von Humboldt Professorship endowed by the German Federal Ministry of Education and Research.

**Conflict of interest:** None declared.

**Key Messages**

- Our study has revealed remarkable geographical variation in HIV incidence in this hyper-endemic population, with the existence of clear ‘corridors of transmission’ where the rate of new HIV infections was 70% higher than surrounding communities.
- Despite the overall high incidence of HIV in many rural African populations, these findings, and observations from similar settings, support an approach that seeks to provide preventive interventions to the most vulnerable geographies as part of an overall combination prevention approach.

## Supplementary Material

Supplementary Data
