# Supplementary material for: Identifying ‘corridors of HIV transmission’ in a severely affected rural South African population: a case for a shift toward targeted prevention strategies
Source: Int J Epidemiol. 2017 Dec 28;47(2):537–49. doi: 10.1093/ije/dyx257 (PMC5913614; doi:10.1093/ije/dyx257)
Supplement: Supplementary Data [file dyx257_ije-2016-12-1547-file008.pdf]

Supplementary material "Identifying corridors of HIV transmission in a severely affected rural South African population: A case for a shift toward targeted prevention strategies"

Frank Tanser, Till Bänighausen, Adrian Dobra, and Benn Sartorius

In this material we describe the statistical models we employed in Section "Cluster characterization." The selected characteristics from Table 1 are compared between clusters and non-clusters based on linear and logistic mixed effects models. We let n be the number of individuals involved, and $n_j \geq 1$ the number of episodes (records) in which the exposure period of individual $j \in \{1, 2, \ldots, n\}$ is divided. We denote by Y_{ij} the value of one of these characteristics for the i-th exposure episode of individual j.

The linear mixed effects models have the following form:

$$Y_{ij} = \mu + \alpha_j + \beta_1 C_{ij}^1 + \beta_2 C_{ij}^2 + \beta_3 C_{ij}^3 + \epsilon_{ij}, \quad (1)$$

$$\epsilon_{ij} \sim \mathsf{N}(0, \sigma_y^2), \quad (2)$$

$$\alpha_j \sim \mathsf{N}(0, \sigma_\alpha^2), \quad (3)$$

for $j = 1, \ldots, n$, and $i = 1, \ldots, n_j$. In Equation (1), $C_{ij}^k = 1$ if individual j was located in cluster $k \in \{1, 2, 3\}$ during the i-th exposure episode; ϵ_{ij} are idiosyncratic errors assumed to be independent and identically distributed between and within individuals, and to follow a Normal distribution (2). The correlation between samples (episodes) associated with the same individual is modeled through the random effects α_j, $j = 1, \ldots, n$, that are assumed to be independent and identically distributed as in (3). Testing whether there is a relationship between a selected characteristic and the clustering of locations is done by testing the null hypothesis:

$$H_0 : \beta_1 = \beta_2 = \beta_3 = 0, \quad (4)$$

against the alternative hypothesis

$$H_A : \beta_1 \neq 0, \text{ or } \beta_2 \neq 0, \text{ or } \beta_3 \neq 0. \quad (5)$$

We note that those selected characteristics that take only positive values (e.g., age) were log-transformed before being used as outcomes in the linear mixed effects model. For a selected characteristic that takes two values $Y_{ij} \in \{0, 1\}$, we employ logistic mixed effects models of the form:

$$\text{logit}\left(\mathsf{P}(Y_{ij} = 1 \mid C_{ij}^1, C_{ij}^2, C_{ij}^3)\right) = \mu + \alpha_j + \beta_1 C_{ij}^1 + \beta_2 C_{ij}^2 + \beta_3 C_{ij}^3, \quad (6)$$

$$\alpha_j \sim \mathsf{N}(0, \sigma_\alpha^2), \quad (7)$$

for $j = 1, \ldots, n$, and $i = 1, \ldots, n_j$. In Equation (6), $\text{logit}(p) = \log \frac{p}{1-p}$, for $p \in (0, 1)$. The cluster membership variables C_{ij}^k, $k = 1, 2, 3$, and the random effects α_j from (7) follow the same definitions and distributional assumptions as for the linear mixed effects model (3). Testing

whether the binary selected characteristic has a relationship with the clustering of locations is also done by testing the null hypothesis (4) against the altenative (5).

We examine the relationship between incidence rates and the clustering of locations by fitting separate Cox proportional hazards models for men and women. We denote by t_{ij} the end time of the i-th episode ($i = 1, \ldots, n_j$) of the exposure period of individual j, $j = 1, \ldots, n$. With this notation, the i-th episode lasts between time $t_{i-1,j}$ and $t_{i,j}$, with $t_{0,j} = 0$. For a time in the i-th exposure episode $t \in [t_{i-1,j}, t_{ij})$, we denote by $\bar{C}_j(t) = \cup_{k=1}^{3} \{C_{i'j}^k : i' \in \{1, 2, \ldots, i\}\}$ the cluster memberships associated with all the episodes that precede the i-th episode, and the i-th episode. We also denote $C_j^k(t) = C_{ij}^k$ if $t \in [t_{i-1,j}, t_{ij})$, for $k = 1, 2, 3$. We specify a Cox proportional hazards model for the time to HIV seroconversion that has the cluster membership indicator variables as time-dependent covariates:

$$\lambda(t \mid \bar{C}_j(t)) = \lambda_0(t) \exp\left(\beta_1 C_j^1(t) + \beta_2 C_j^2(t) + \beta_3 C_j^3(t)\right), \tag{8}$$

where $\lambda(t \mid \bar{C}_j(t))$ is a conditional hazard function, and $\lambda_0(t)$ is an unspecified baseline hazard function. Testing whether the clustering of locations is associated with the hazard of HIV acquisition is performed by testing the null hypothesis (4) against the alternative hypothesis (5) in the Cox proportional hazards model (8).
